# Associations of passive muscle stiffness, muscle stretch tolerance, and muscle slack angle with range of motion: individual and sex differences

**DOI:** 10.1038/s41598-018-26574-3

**Published:** 2018-05-29

**Authors:** Naokazu Miyamoto, Kosuke Hirata, Eri Miyamoto-Mikami, Osamu Yasuda, Hiroaki Kanehisa

**Affiliations:** Department of Sports and Life Science, National Institute of Fitness and Sports in Kanoya, 1 Shiromizu, Kanoya, Kagoshima, 891-2393 Japan

## Abstract

Joint range of motion (ROM) is an important parameter for athletic performance and muscular injury risk. Nonetheless, a complete description of muscular factors influencing ROM among individuals and between men and women is lacking. We examined whether passive muscle stiffness (evaluated by angle-specific muscle shear modulus), tolerance to muscle stretch (evaluated by muscle shear modulus at end-ROM), and muscle slack angle of the triceps surae are associated with the individual variability and sex difference in dorsiflexion ROM, using ultrasound shear wave elastography. For men, ROM was negatively correlated to passive muscle stiffness of the medial and lateral gastrocnemius in a tensioned state and positively to tolerance to muscle stretch in the medial gastrocnemius. For women, ROM was only positively correlated to tolerance to muscle stretch in all muscles but not correlated to passive muscle stiffness. Muscle slack angle was not correlated to ROM in men and women. Significant sex differences were observed only for dorsiflexion ROM and passive muscle stiffness in a tensioned state. These findings suggest that muscular factors associated with ROM are different between men and women. Furthermore, the sex difference in dorsiflexion ROM might be attributed partly to that in passive muscle stiffness of plantar flexors.

## Introduction

Joint flexibility is an important component of physical fitness that is generally believed to influence muscular injury risk^1,2^ and athletic performances^3,4^. Joint flexibility is usually quantified by measuring a range of motion (ROM) about the joint^5–8^, and ROM measurements are common in medical and rehabilitation fields when making diagnoses, setting treatment goals, and evaluating treatment progress^9^. Thus, it is important to elucidate the factors affecting ROM.

With respect to individual variability in ROM, Magnusson *et al*.^10^ have shown that individuals with greater ROM (determined by the onset of pain) exhibited higher passive torque at end-ROM than those with smaller ROM. Based on this, they suggested that individual variability in ROM is related to that in sensation to pain and/or muscle stretch (often referred to as stretch tolerance). Additionally, they reported that individuals with greater ROM had less stiff muscle, based on the results of the comparison of passive joint torque-joint angle relationship between flexible and inflexible individuals. Collectively, both individual variabilities in passive muscle stiffness and tolerance to muscle stretch have been considered to be responsible for that in ROM^8,10–13^. However, it should be noted that passive torque is a measure related to the resistance of the entire musculo-articular complex, and involves several anatomical structures crossing the joint. Namely, it is impossible to assess passive muscle stiffness and tolerance to muscle stretch from the findings on the passive torque-angle relationship. Thus, it remains unclear how the individual variabilities in passive muscle stiffness and tolerance to muscle stretch are related to that in ROM.

In addition to individual variability, the sex difference in joint flexibility has been examined. Women have been shown to exhibit a greater ROM than men^5,14,15^, while women have greater sensitivity and lower tolerance to pain than men^16,17^. Thus, it is expected that passive muscle stiffness is lower in women than in men since lower passive muscle stiffness and higher tolerance to muscle stretch can be responsible for increased ROM as mentioned above. However, the underlying mechanisms for the sex difference in ROM are not well understood. Furthermore, there is a possibility that the associations of passive muscle stiffness and muscle stretch tolerance to ROM vary between men and women. However, little is still known about how the factors affect ROM by sex.

One approach to evaluate stiffness and tension of individual muscles is the use of ultrasound shear wave elastography (SWE). This is a recently developed imaging technique that can quantify localized tissue shear modulus, based on the propagation speed of remotely induced shear wave^18,19^. Furthermore, the use of SWE enables to detect a slack angle of a muscle (i.e., the joint angle beyond which the muscle begins to develop passive tension)^20–22^. Theoretically, individuals with greater slack angles of muscles can exhibit greater ROM under the same muscle stress even when passive muscle stiffness is same. An acute bout of stretching exercise has been shown to increase ROM in association with the increase of muscle slack angle^23,24^. Thus, it is expected that individuals with greater muscle slack angle exhibit greater ROM. To date, no study has examined whether the differences in muscle slack angle among individuals and between sexes are related to those in ROM. If slack angle of a muscle is the primary determinant of ROM, ROM assessment may be useless despite the facts that ROM assessment has often been clinically used to assess muscle stiffness/extensibility and that ROM is believed to relate to muscular injury risk.

Therefore, taking advantage of SWE, this study aimed to elucidate muscular factors affecting individual variability and sex difference in ROM, from the viewpoint of passive muscle stiffness, tolerance to muscle stretch, and muscle slack angle. As mentioned above, we hypothesized that the factor(s) associated with ROM would vary between men and women. The second hypothesis was that passive muscle stiffness would be lower in women than in men, leading to greater ROM in women.

## Results

### Experiment 1

**Figure 1:**
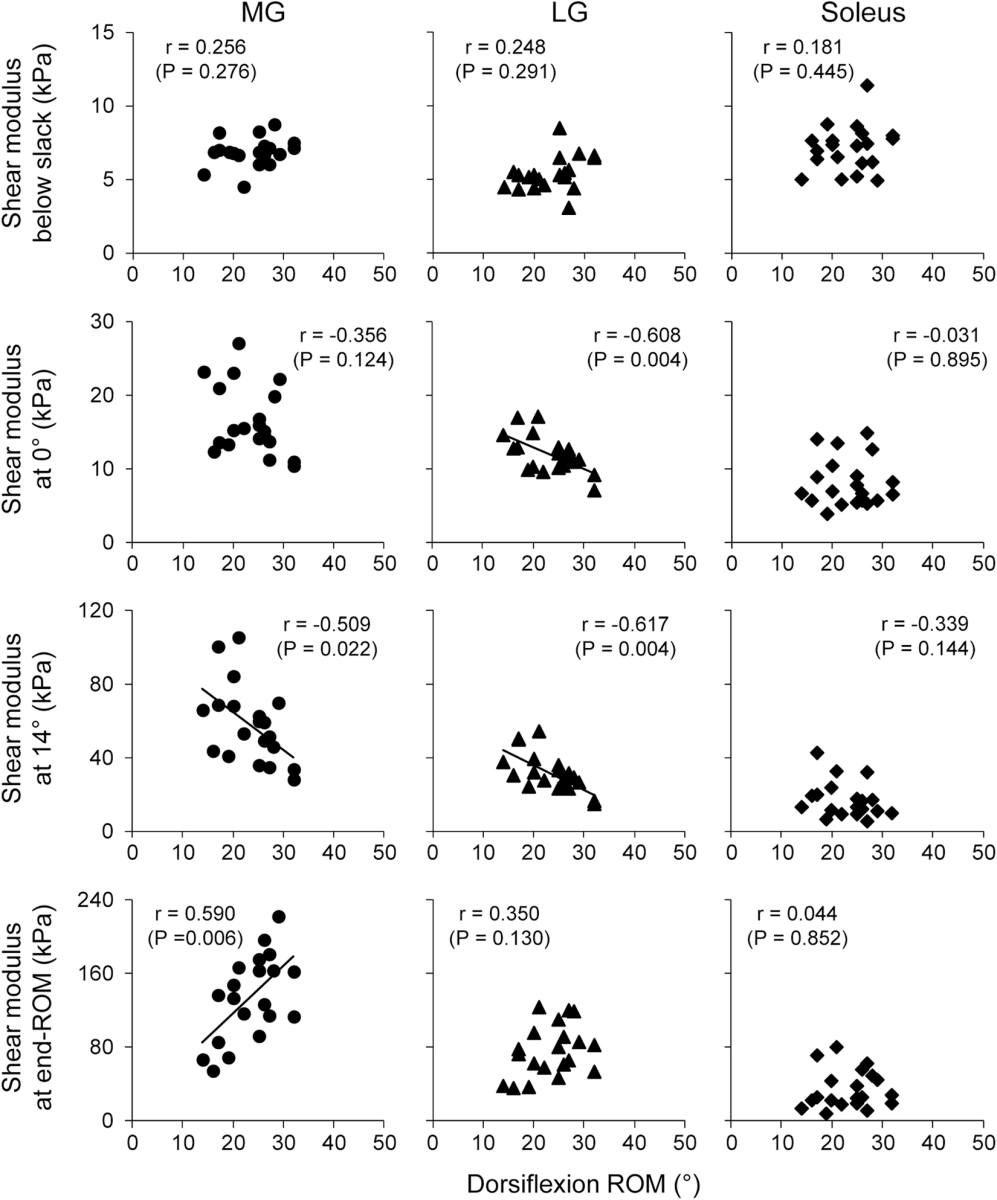
Relationships between dorsiflexion range of motion (ROM) and passive muscle stiffness (evaluated by angle-specific shear modulus) or tolerance to muscle stretch (evaluated by shear modulus at end-ROM) of the medial (MG) and lateral gastrocnemius (LG), and soleus in men.

**Figure 2:**
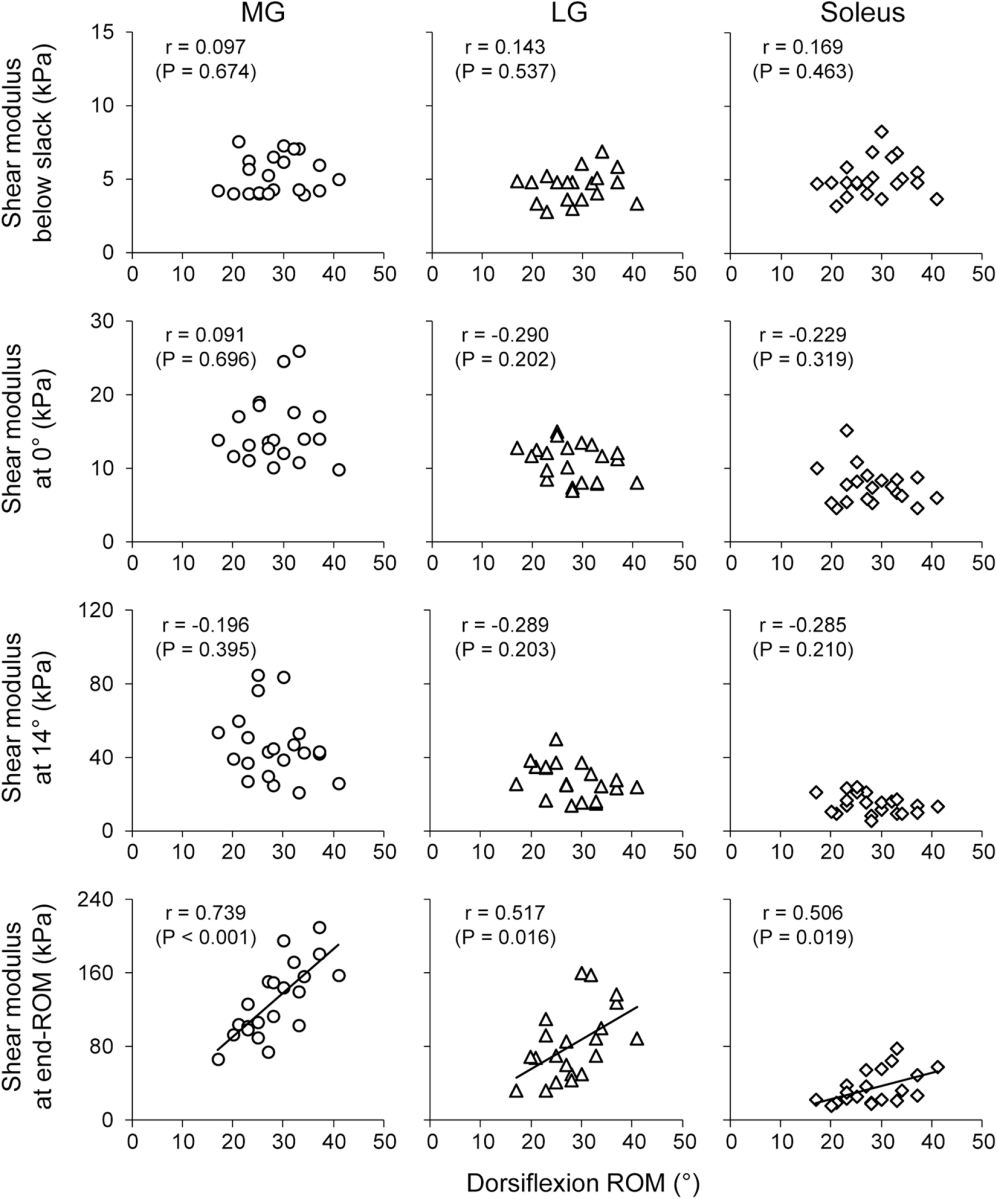
Relationships between dorsiflexion range of motion (ROM) and passive muscle stiffness (evaluated by angle-specific shear modulus) or tolerance to muscle stretch (evaluated by shear modulus at end-ROM) of the medial (MG) and lateral gastrocnemius (LG), and soleus in women.

Figures 1 and 2 show the relationships between dorsiflexion ROM and angle-specific muscle shear modulus (i.e., passive muscle stiffness) or muscle shear modulus at end-ROM (i.e., tolerance to muscle stretch) in each muscle for men and women, respectively. For men (Fig. 1), dorsiflexion ROM was negatively correlated to LG shear modulus at 0° (*r* = −0.608, *P* = 0.004), to MG and LG shear modulus at 14° (*r* = −0.509, *P* = 0.022 for MG; *r* = −0.617, *P* = 0.004 for LG), and positively to MG shear modulus at end-ROM (*r* = 0.509, *P* = 0.006). For women (Fig. 2), dorsiflexion ROM was positively correlated to muscle shear modulus at end-ROM of all muscles (*r* = 0.739, *P* < 0.001 for MG; *r* = 0.517, *P* = 0.016 for LG; *r* = 0.506, *P* = 0.019 for Sol), but not to angle-specific muscle shear modulus. Additionally, dorsiflexion ROM was not correlated to slack angle of each muscle for men and women.

Table 1 shows dorsiflexion ROM, angle-specific muscle shear modulus, muscle shear modulus at end-ROM, and slack angle of each muscle in men and women. ROM was significantly greater in women than in men (*d* = 0.87, *P* < 0.001). For muscle shear modulus below the slack angle, shear modulus at 0°, and shear modulus at end-ROM, two-way ANOVAs revealed only a significant main effect of muscle factor (slack: *F* = 13.119, *P* < 0.001, partial η^2^ = 0.252; 0°: *F* = 76.633, *P* < 0.001, partial η^2^ = 0.663; end-ROM: *F* = 207.601, *P* < 0.001, partial η^2^ = 0.842) without muscle × sex interaction. According to post-hoc Bonferroni tests, muscle shear modulus below the slack angle was significantly greater in the MG and soleus than in LG (MG vs. LG: *d* = 0.87, *P* < 0.001; soleus vs. LG: *d* = 0.77, *P* < 0.001). Muscle shear modulus at 0° was significantly greater in order of MG > LG > soleus (MG vs. LG: *d* = 1.16, *P* < 0.001; LG vs. soleus: *d* = 1.29, *P* < 0.001; MG vs. soleus: *d* = 2.03, *P* < 0.001). Muscle shear modulus at end-ROM was significantly greater in order of MG > LG > soleus (MG vs. LG: *d* = 1.39, *P* < 0.001; LG vs. soleus: *d* = 1.65, *P* < 0.001; MG vs. soleus: *d* = 2.98, *P* < 0.001). For muscle shear modulus at 14°, there was a significant muscle × sex interaction (*F* = 3.224, *P* = 0.045, partial η^2^ = 0.076). According to post-hoc Bonferroni tests, the muscle shear modulus at 14° was significantly greater for both men and women in order of MG > LG > soleus (men: MG vs. LG: *d* = 1.58, *P* < 0.001; LG vs. soleus: *d* = 1.58, *P* < 0.001; MG vs. soleus: *d* = 2.53, *P* < 0.001; women: MG vs. LG: *d* = 1.49, *P* < 0.001; LG vs. soleus: *d* = 1.71, *P* < 0.001; MG vs. soleus: *d* = 2.60, *P* < 0.001). Post-hoc unpaired t-tests revealed that muscle shear modulus at 14° of MG and LG was significantly greater in men than in women (MG: *d* = 0.74, *P* < 0.001; LG: *d* = 0.70, *P* < 0.001). For muscle slack angle, there was only a significant main effect of muscle factor (*F* = 0.294, *P* < 0.001, partial η^2^ = 0.508) without muscle × sex interaction. A post-hoc Bonferroni test revealed that the slack angles in MG and LG were significantly smaller (i.e., more plantar flexed position) than that in the soleus (soleus vs. MG: *d* = 1.46, *P* < 0.001; soleus vs. LG: *d* = 1.37, *P* < 0.001).

**Table 1:** Dorsiflexion range of motion, shear modulus, and slack angle in men and women.

	Men (n = 20)	Women (n = 21)
ROM (°)**	23.4 ± 5.2	28.3 ± 6.3
Shear modulus (kPa)		
Below slack		
MG	6.9 ± 1.0	6.3 ± 1.3
LG	5.4 ± 1.2	4.9 ± 1.0
Soleus	7.1 ± 1.6	6.3 ± 1.2
At 0°		
MG	16.4 ± 4.7	14.8 ± 4.4
LG	11.9 ± 2.5	10.7 ± 2.5
Soleus	8.0 ± 3.3	7.5 ± 2.5
At 14°^†^		
MG*	58.0 ± 21.1	44.6 ± 15.9
LG*	32.0 ± 10.0	25.7 ± 8.3
Soleus	16.5 ± 9.6	14.0 ± 5.0
At end-ROM		
MG	134.0 ± 45.8	130.0 ± 39.8
LG	75.4 ± 28.1	82.2 ± 38.4
Soleus	33.6 ± 20.7	34.4 ± 17.8
Slack angle (degree)		
MG	−22.1 ± 7.4	−21.6 ± 6.1
LG	−21.0 ± 8.2	−22.5 ± 8.2
Soleus	−6.1 ± 12.3	−7.4 ± 13.0

*Significantly different between men and women (P < 0.05).

**Significantly different between men and women (P < 0.01).

^†^Significant muscle × sex interaction (P < 0.05).

LG: lateral gastrocnemius, MG: medial gastrocnemius, ROM: range of motion.

For RMS-EMG data, no main effects or interactions were observed, with values across muscles of 1.6 ± 0.9% and 1.8 ± 0.7% MVC at end-ROM for men and women, respectively, indicating that the dorsiflexion measurements were performed under passive conditions for men and women.

### Experiment 2

Table 2 shows ROM, serum estradiol and progesterone levels, MG shear modulus, and MG slack angle in the menstrual, ovulatory, and luteal phases. For ROM, one-way ANOVAs revealed no significant main effect of phase factor. Serum hormonal levels were within the ranges reported for normal, healthy women. The estradiol concentrations were significantly lower during the menstrual phase than during the other phases (ovulatory vs. menstrual: *d* = 2.54, *P* = 0.003; luteal vs. menstrual: *d* = 2.54, *P* = 0.001). For the progesterone, the concentration during the luteal phase was significantly higher than those during the other phases (luteal vs. menstrual: *d* = 3.98, *P* < 0.001; luteal vs. ovulatory: *d* = 3.72, *P* = 0.001). For MG muscle shear modulus at each angle and slack angle, one-way ANOVAs revealed no significant main effect of phase factor (*d* ≤ 0.321, *P* > 0.405).

**Table 2:** Serum hormonal levels and muscle properties during the menstrual, ovulatory, and luteal phases.

	Phase		
	Menstrual	Ovulatory	Luteal
ROM (°)	25.8 ± 6.4	25.6 ± 5.1	28.1 ± 5.8
Estradiol (pg/ml)	33.1 ± 11.6	165.5 ± 72.7*	153.9 ± 46.2*
Progesterone (ng/ml)	0.90 ± 0.33	1.29 ± 1.12	12.40 ± 4.07*^†^
MG shear modulus (kPa)			
Below slack	3.4 ± 1.4	3.2 ± 1.0	3.9 ± 1.3
At 0°	12.2 ± 3.5	13.4 ± 2.4	13.2 ± 4.9
At 13°	47.1 ± 28.2	47.4 ± 10.5	47.5 ± 11.3
At end-ROM	97.7 ± 28.4	107.4 ± 32.0	98.5 ± 31.7
MG slack angle (degree)	−21.1 ± 5.4	−22.6 ± 6.6	−20.9 ± 5.7

MG: medial gastrocnemius, ROM: range of motion.

*Significantly different from menstrual phase.

^†^Significantly different from ovulatory phase.

## Discussion

As mentioned in the earlier section, it is impossible to assess passive muscle stiffness and tolerance to muscle stretch from the findings on the passive torque-angle relationship. To overcome these limitations, in the present study, passive muscle stiffness and tolerance to muscle stretch were evaluated by measuring angle-specific muscle shear modulus and muscle shear modulus at end-ROM, respectively. Regarding the latter, although we did not assess the region where participants felt pain (i.e., muscle or tendon), the tendon elongation during passive dorsiflexion is smaller than that during plantar flexion MVC^25^. Thus, it is reasonable to suppose that participants felt pain in the stretched muscles rather than the stretched tendons and that the individual variability in muscle shear modulus at end-ROM eventually indicates the variability in tolerance to muscle stretch.

In men, dorsiflexion ROM was negatively correlated to passive muscle stiffness at 0° (with a large effect size of *r* = −0.608 in LG) and 14° (with a large effect size of *r* = −0.509 and −0.617 in MG and LG, respectively) but not to passive muscle stiffness below the slack angle (small effect size in all muscles). These findings suggest that, in men, passive muscle stiffness in a tensioned state, not in a slack state, is related to ROM. Additionally, dorsiflexion ROM was positively related to tolerance to muscle stretch in MG (with a large effect size of *r* = −0.590). Considering these findings together, the present findings in men strongly support the indications of the previous studies^10,11^ that the individual variability in ROM is attributed to those in both passive muscle stiffness and tolerance to muscle stretch although the cause and effect relationship is not clear. In contrast to the case in men, dorsiflexion ROM was not correlated to passive muscle stiffness in women (with small effect sizes of absolute *r* ≤ 0.290). One might doubt that this finding in women is due to the joint angle measured for the passive muscle stiffness. We, therefore, performed additional analysis to examine the robustness of our results by evaluating the passive muscle stiffness at the smallest dorsiflexion ROM of the 21 women participants (17°). The additional analyses showed similar results to those above (absolute *r* ≤ 0.324, *P* ≥ 0.152), confirming the present results and conclusions.

When comparing ROM between the sexes, women exhibited greater ROM than men (with a large effect size of *d* = 0.87) in the present study. This is in agreement with the previous findings^5,14,15^. The only other significant difference between men and women was found for the passive muscle stiffness at 14° of MG and LG (with medium effect sizes of *d* = 0.74 and 0.70 in MG and LG, respectively). This is inconsistent with a recent study which failed to find the sex difference in passive muscle stiffness of MG evaluated using SWE^26^. The following three reasons could explain the discrepancy between the present and previous studies. First, the muscle thickness of MG was similar between men and women in the previous study whereas muscle thickness in females was significantly smaller than that in males in the present study (*d* = 1.62, *P* < 0.001) (although data not shown). Since it has been well documented that, regardless of limbs, muscle size (e.g., cross-sectional area and thickness) is generally larger in men than in women, the previous findings cannot be generalized to universal statements about the sex difference in muscle stiffness. Second, the EMG activity during passive dorsiflexion was not measured in the previous study. Thus, it remains unclear whether the magnitude of relaxation of the muscle was comparable between men and women in the previous study. Third, in the present study, in order to minimize or avoid the effect of inevitable joint angular displacement (which results in the discrepancy between the movements of ankle joint and footplate of the dynamometer) on the observed muscle shear modulus due to changes in muscle length, the ankle joint angles referred to the angle assessed with the goniometer instead of the angle of the dynamometer. In contrast, in the previous study, the joint angles referred to the angle of the dynamometer, in which the inevitable joint angular displacement was not considered. Anyway, considering the present findings together, it is suggested that the sex difference in ROM is associated with that in passive muscle stiffness in a tensioned state. Although it may seem insufficient or incorrect from a statistical perspective, it is reasonable from a mechanical viewpoint to consider that the stiffest muscle is the primary determining factor for ROM. MG exhibits the highest passive muscle stiffness among the triceps surae, which is in agreement with previous findings^24^. Collectively, the sex difference in dorsiflexion ROM could be strongly related to that in MG stiffness. On the other hand, nerve and/or fascia have recently been proposed to influence ROM^27–29^. Further researches are required to determine the limiting factor for ROM.

The intramuscular connective tissue (consisting mainly of collagen), particularly the perimysium, is considered to be a major extracellular contributor to passive muscle stiffness^11,30,31^. Thus, the concentration of such intramuscular connective tissue might differ between men and women, leading to the sex difference in passive muscle stiffness in a tensioned state observed in the present study. To our knowledge, however, this remains unknown. Another possible mechanism for the sex difference in passive muscle stiffness is the influence of sex hormonal levels. For example, oestrogen has been reported to alter structural and mechanical properties of collagenous tissues; including decreased collagen synthesis and increased degradation^32,33^. Oestrogen receptors have been identified within human skeletal muscle^34,35^. Thus, it appears likely that passive muscle stiffness is influenced by sex hormonal levels. In Experiment 2, however, we failed to find significant effects of the menstrual cycle phases and corresponding hormonal levels on passive muscle stiffness even in a tensioned state, suggesting that the results of Experiment 1 are not influenced by the menstrual cycle phases of the women participated. Moreover, although serum hormone levels were not measured in men of the present study, the serum estradiol-β−17 concentration of healthy young men reported in previous studies (e.g., 24.8 ± 8.4 pg/ml^36^) is not so different from that during the menstrual phase in Experiment 2 of the present study. Taking these observations together, the effect of oestrogen on passive muscle stiffness, if any, might not be acute but an accumulated effect over several cycles.

Additionally, the sex difference in tendon mechanical properties might be related to that in the passive muscle stiffness of the present study. Kubo *et al*.^37^ reported the sex difference in tendon mechanical properties such as stiffness and Young’s modulus. However, careful attention must be paid when considering the previous findings. In the previous study, tendon stiffness and Young’s modulus, which were calculated based on the measurements of tendon elongation under high tendon force (stress) conditions (above 50% MVC, i.e., linear region), were significantly higher in young men than in young women. In contrast, tendon elongation and strain under low tendon force/stress conditions as in the present study were reported to be not significantly different between men and women^37^, implying that tendon mechanical properties under low tendon stress conditions are comparable between men and women. Therefore, it is likely that the effect of sex difference in tendon mechanical properties, if any, on the present experimental results is small.

At the start of the present study, it was expected that muscles with greater slack angles would result in greater ROM. However, contrary to the expectation, the individual variability of muscle slack angle was not related to that in ROM in both men and women, and there was no significant difference between the sexes regardless of the sex difference in ROM. A recent study has shown that the increase in dorsiflexion ROM after an acute bout of static stretching was associated with the increase of muscle slack angle of the triceps surae^24^. Collectively, the effect of individual variability in muscle slack angle on that in ROM may be negligibly small.

In conclusion, the primary purpose of the present study was to examine whether passive muscle stiffness, muscle stretch tolerance, and muscle slack angle of the triceps surae are associated with the individual variability and sex difference in dorsiflexion ROM. The findings obtained here suggest that muscular factors associated with ROM are different between men and women. Specifically, in men, the individual variability in ROM is related to that in passive muscle stiffness and tolerance to muscle stretch. In contrast, in women, the individual variability in ROM is associated with that in tolerance to muscle stretch but not passive muscle stiffness. The sex difference in dorsiflexion ROM might be attributed to that in passive muscle stiffness of MG, although the cause and effect relationship is presently not clear. Furthermore, it is indicated that passive muscle stiffness, tolerance to muscle stretch, and muscle slack angle in women are not influenced by the menstrual cycle phases. The association between joint flexibility and risk of muscular injuries such as muscle strain in sports has been debated over several years. Some studies have reported that smaller ROM increases the risk of the muscle strain, whereas women display a greater ROM of ankle dorsiflexion and a greater risk of muscular injuries at the ankle and calf compared to men. As an explainable reason for the discrepancy, we expected the difference in factors determining the maximum ROM between men and women, based on the present findings. The present findings for women can be evidence for the background of greater risk of muscular injuries in women. Additionally, measures of maximum dorsiflexion ROM partly reflect muscle stiffness of the triceps surae only in men. Thus, clinicians should use another functional flexibility test of the ankle joint when assessing muscle stiffness of the plantar flexors in women.

## Methods

In the present study, on the basis of the results of the primary experiment (Experiment 1) which was performed to achieve the aforementioned objective, an additional experiment (Experiment 2) was designed to investigate the effect of the menstrual cycle phases on passive muscle stiffness, tolerance to muscle stretch, and muscle slack in women. As mentioned in the earlier section, passive muscle stiffness and tolerance to muscle stretch were evaluated by measuring angle-specific muscle shear modulus and muscle shear modulus at end-ROM, respectively. In both experiments, all participants were healthy young adults and had no apparent neurological, orthopaedic, or neuromuscular problems. They were asked to refrain from strenuous exercise 24 h before the experiments. All participants gave written informed consent prior to participation. This study was approved by the ethics committee of National Institute of Fitness and Sports in Kanoya and performed in accordance with the Declaration of Helsinki.

### Experiment 1

#### Participant

Twenty men (172.7 ± 4.6 cm, 65.9 ± 3.4 kg, 21.6 ± 3.1 years; mean ± SD) and 21 women (160.8 ± 4.4 cm, 55.4 ± 6.7 kg, 21.4 ± 2.1 years) participated in Experiment 1.

#### Experimental setup and procedure

The procedures for measuring ankle joint angle, muscle shear modulus, and electromyographic (EMG) activity were the same as those used in our previous studies^23,24^. Participants lay on a dynamometer (CON-TREX MJ, PHYSIOMED, Germany) bed in a prone position with their right knee fully extended. The right foot was firmly secured to the dynamometer’s footplate. The rotation axes of the footplate and ankle were aligned as closely as possible. The ankle joint was passively dorsiflexed from 50° plantar flexion (anatomically neutral position defined as 0°, with positive values for dorsiflexion) to the maximum dorsiflexion angle which was defined as the onset of pain in the stretched musculotendinous tissues for each participant, at an angular velocity of 1°/s. This angular velocity was adopted to obtain a better resolution for the SWE measurements (see below) and to avoid or minimize the stretch reflex^20,21,23,24,38^, which could stiffen the muscles. The participants were requested to completely relax and not to resist the movement of the footplate throughout the passive dorsiflexion. Firstly, in order to familiarize the participants to the procedure, to ensure that they were as relaxed as possible, and to avoid bias related to a conditioning effect, 2 cycles of the passive dorsiflexion (familiarization session) was performed to the maximum dorsiflexion angle which was determined as the onset of pain during the respective cycles^13,20,23,24^. Then, the testing session was performed. Namely, the measurements were performed during the 3rd cycle and later. The maximum dorsiflexion angle determined in the 1st cycle of the testing session was used for the subsequent cycles of the testing session. Passive ankle dorsiflexion was stopped when participants pressed a stop button, which was followed by returning the ankle joint angle to a plantar flexed position immediately and automatically in order to avoid a stretching effect. The angular displacement of the ankle joint was measured with a goniometer (SG110/A, Biometrics, UK) fixed to the ankle joint. In the present study, all reported angles refer to the ankle joint angle assessed with the goniometer, not the angle of the dynamometer footplate.

#### SWE and EMG

Two ultrasound SWE systems (Aixplorer Ver.6 and 8, Supersonic Imagine, France), each coupled with a linear array probe (SL15-4, Supersonic Imagine, France), were used in SWE mode (MSK preset; persistence = Med, smoothing = 5) to quantify the muscle shear modulus for each of the medial (MG) and lateral gastrocnemius (LG), and soleus. The validity and repeatability of the muscle SWE measurement have been proven in phantom and human experiments in our previous reports^24,39,40^. SWE measurements were performed over the muscle belly (i.e., at approximately 30–40% of the lower leg length) for MG and LG, and medial or lateral aspect distal to the gastrocnemius muscle-tendon junction for Sol, according to our previous studies^20,24^. The probe orientation was adjusted to identify fascicles within the B-mode image in each muscle. Then, the passive dorsiflexion was initiated after the examiners recognized the colour map was stable for a few seconds. The probe location was adjusted slightly prior to image acquisition if a defocused image with a large variation in the colour map was observed. Care was taken not to press and deform the muscles while scanning. Since three muscles were targeted with two SWE systems, the passive dorsiflexion was repeated over the same ROM (determined in the 1st measurement of the testing session) for assessing the other muscle. The order of SWE measurements for the MG, LG, and soleus was randomly assigned and counterbalanced across participants.

After the determination of the probe location, pre-amplified active surface EMG electrodes (1 mm width × 8 mm length, inter-electrode distance: 12 mm) with band-pass filtering between 5 and 450 Hz (FA-DL-141, 4-assist, Japan) were placed just medial or lateral to each ultrasound probe position of the MG, LG, and soleus.

#### Data analysis

**Figure 3:**
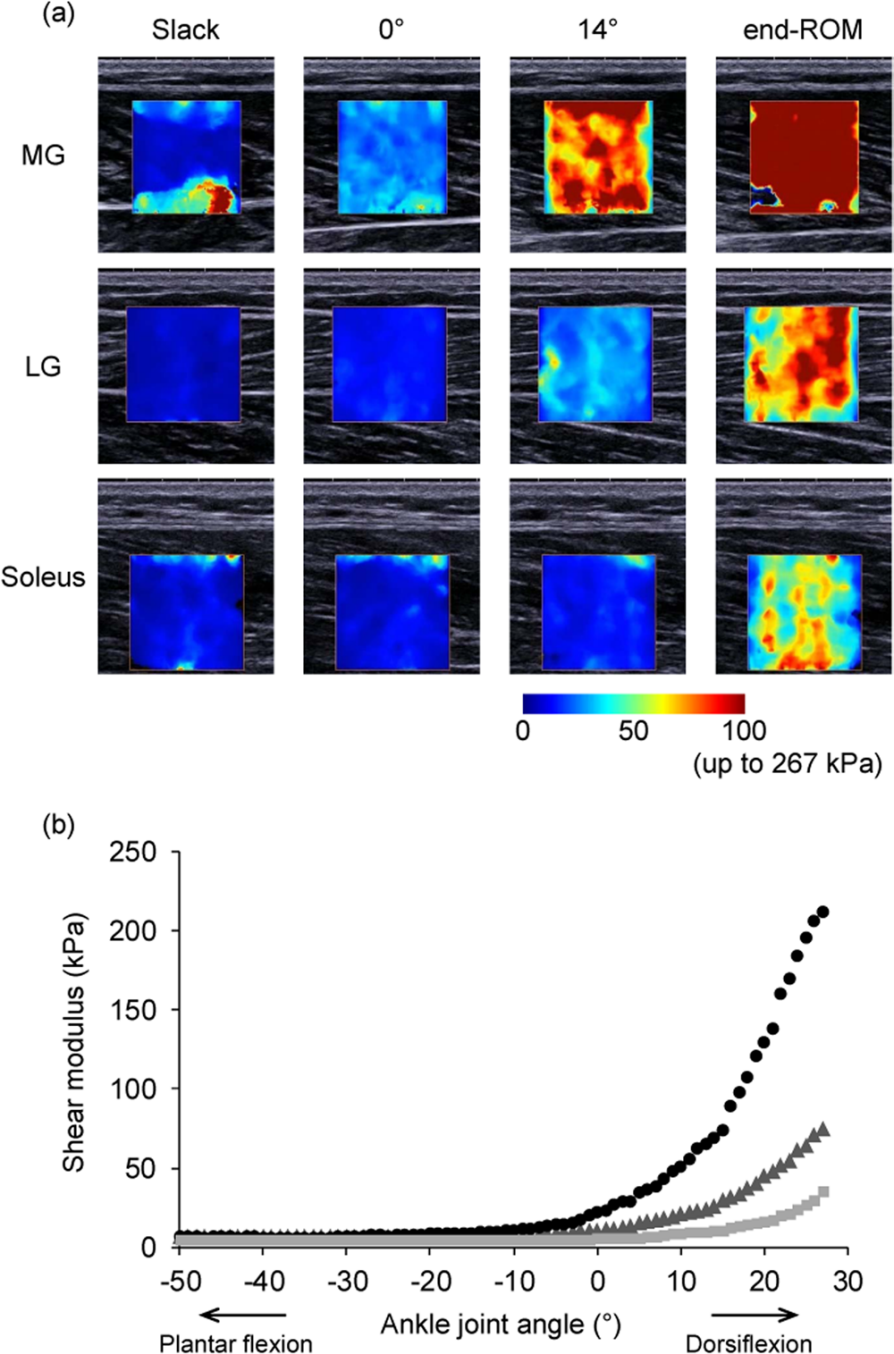
The upper images (**a**) are typical examples of ultrasound shear wave elastography measurements of the medial (MG) and lateral gastrocnemius (LG) and soleus obtained at slack angle, 0°, and 14° dorsiflexion angle and at end of dorsiflexion range of motion (end-ROM). The coloured region represents the shear modulus map with the scale below the images. The lower graph (**b**) shows an example of the responses in shear modulus of MG (black circle), LG (dark grey triangle), and soleus (light grey square) during passive dorsiflexion. Note that no value in the region of interest reached the saturation limit (267 kPa) while the upper limit of the examples is set at 100 kPa instead of 267 kPa for clarity.

The joint angle and EMG signals were simultaneously recorded at 1 kHz by using a 16-bit analogue-to-digital converter (PowerLab 16/35, ADInstrument, Australia) and manually synchronized with SWE recording (sampling frequency = 1 Hz, that is, one image per angle; Fig. 3). For SWE data, processing was performed by software of the ultrasound SWE system. A circular area as large as possible with the exclusion of subcutaneous adipose tissues and aponeuroses for each image (the size being dependent on the participant and muscle) was selected as the region of interest for shear modulus calculation^20,23,24,39^. No value in the circular region reached the saturation limit of shear modulus for SWE system (267 kPa). In Experiment 1, since the smallest dorsiflexion ROM of the total 41 participants was 14° (in a man), passive muscle stiffness at 0° and 14° was evaluated. Additionally, according to previous studies^20,23,24,41^, the slack angle of each muscle was visually determined from the ankle joint angle-shear modulus relationship as the first increase above the variation in shear modulus by an experienced examiner. Then, passive muscle stiffness below the slack angle was also calculated by averaging data between −50° and slack angle for each muscle. For EMG data, the root-mean-square values (RMS-EMG) were calculated over 250 ms period, during which there was no noise originating from ultrasound SWE scanner, at each joint angle for each muscle. Then, the RMS-EMG values of each muscle were normalized to that obtained during maximal voluntary isometric contraction (%MVC) which was performed at the ankle angle of 0° following the SWE measurements.

#### Statistics

With reference to data of our preliminary results (n = 12), priori power analyses with an assumed type 1 error of 0.05 and a statistical power of 80% were conducted to find statistically significant correlations between dorsiflexion ROM and MG passive muscle stiffness or tolerance to muscle stretch in MG. The critical sample sizes were estimated to be 15 and 9 for the correlations, respectively. Thus, approximately 20 participants were recruited to account for possible attrition in each sex.

Pearson’s correlation analyses were performed between dorsiflexion ROM and explanatory variables (i.e., passive muscle stiffness, tolerance to muscle stretch, muscle slack angle) separately for each sex data. For passive muscle stiffness at each joint angle (i.e., slack, 0°, and 14°), tolerance to muscle stretch, RMS-EMG at each joint angle, and slack angle, two-way analyses of variance (ANOVAs) (muscle × sex) with repeated measures on one factor (muscle) were performed. If appropriate, post-hoc Bonferroni analyses and unpaired t-test were used for comparisons between three muscles and between sexes, respectively. The significant level for all comparisons was set at P < 0.05. When the results of t-test, ANOVA, and Pearson’s correlation are presented, Cohen’s d, partial η^2^, and correlation coefficient (r) are shown as indices of effect size with P value, respectively. The effect size was interpreted as small (d > 0.20, absolute r > 0.10), medium (d > 0.50, absolute r > 0.30), and large (d > 0.80, absolute r > 0.50). All the statistical analyses were performed with statistical software (SPSS Statistics 22, IBM Japan, Japan). Data are expressed as means and SDs.

### Experiment 2

#### Participants

Eight healthy young women (160.4 ± 4.8 cm, 57.6 ± 8.8 kg, 23.9 ± 3.1 years) who were experiencing normal menstrual cycles (reported 28–33 day cycles for the last 6 months) participated in Experiment 2. All participants were either sedentary or recreationally active women, but not were involved in any type of exercise training program during the period of Experiment 2. They had not taken oral hormonal contraceptives or other forms of hormones for at least 2 years prior to and during the study.

#### Testing schedule

Each participant was tested in three phases of her menstrual cycle: menstrual phase (1–3 days after menstruation; when estradiol and progesterone concentrations are low), ovulatory phase (1–2 days before or on predicted ovulation day; when estradiol is elevated and progesterone is low), and luteal phase (7–10 days after predicted ovulation; when estradiol and progesterone are elevated). Using the menstrual cycle data obtained from the questionnaire, the length of menstrual cycle for each participant was calculated by averaging the cycle length of her the latest 6 cycles, and thereby the beginning of the next cycle could be estimated. Then, the date of ovulation was predicted by counting back 14 days from the presumed first day of the next cycle. The order of testing was randomized across participants and the three tests were conducted at the same time of day to minimize any influences of circadian rhythm.

#### Experimental procedure and data analysis

The experimental setup and procedure of measuring ankle joint angle and SWE were the same as those used in Experiment 1, except for the muscles studied with SWE (only MG in Experiment 2). In Experiment 2, since the smallest dorsiflexion ROM of the total 24 tests (8 participants × 3 phases) was 13°, the passive stiffness at 0° and 13° was evaluated.

#### Blood sample

Venous blood samples (~5 ml) were taken from an antecubital vein by a medical practitioner using vacutainers to measure the concentrations of estradiol-β−17 and progesterone. Serum samples were obtained by centrifuging the blood samples at 2900 × g for 10 min at 4 °C, and then stored at −80 °C until analysis. The serum estradiol-β−17 and progesterone concentrations were measured at the Clinical Pathology Laboratory in Kagoshima, Japan.

#### Statistics

For each variable, one-way repeated measure ANOVA was performed to compare between the three phases. If appropriate, post-hoc Bonferroni analyses were used.
